# Supplementary material for: Efficacy of anti-CD147 chimeric antigen receptors targeting hepatocellular carcinoma
Source: Nat Commun. 2020 Sep 23;11:4810. doi: 10.1038/s41467-020-18444-2 (PMC7511348; doi:10.1038/s41467-020-18444-2)
Supplement: Supplementary file 1 — Supplementary Information [file 41467_2020_18444_MOESM1_ESM.docx]

**Supplemental Information**

**Efficacy of anti-CD147 Chimeric Antigen Receptors Targeting Hepatocellular Carcinoma**

Tseng et al.

**Supplementary Figure 1**

**
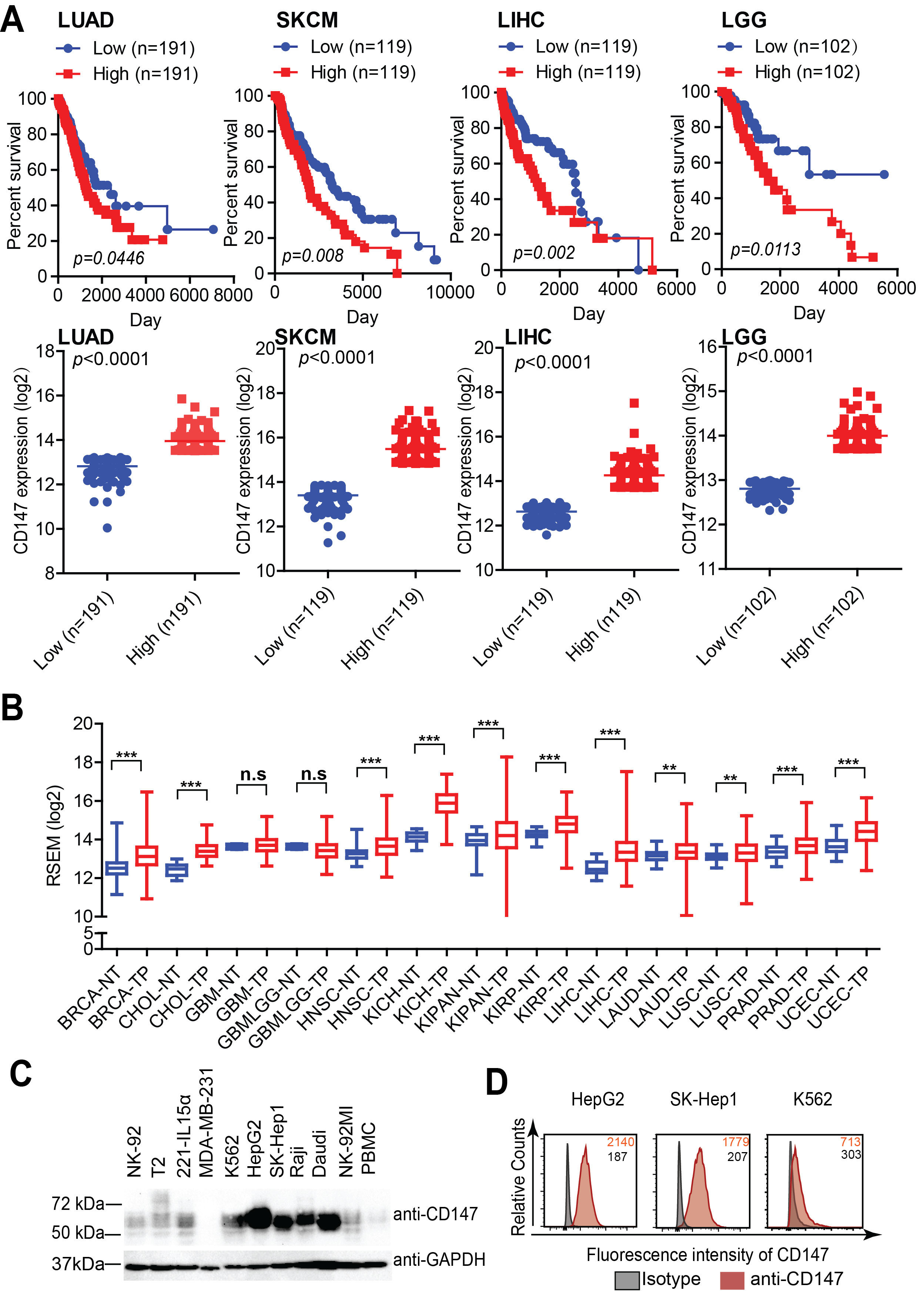
**

**Supplementary Figure 1: CD147 overexpression in hepatocellular carcinoma cells.** (**A**) Prognostic value of upregulated CD147 expression for overall survival of human cancer patients from TCGA datasets. Survival curves (top panel) of 2 different patient populations based on relative CD147 high and low expressions (below panel). The data of LUAD (Lung adenocarcinoma), SKCM (Skin Cutaneous Melanoma), LIHC (Liver hepatocellular carcinoma) and LGG (Brain Lower Grade Glioma) are collected for analysis. (**B**) Comparison of CD147 expression between normal tissue (NT) and tumor patient (TP) sample in multiple cancer types from TCGA datasets. Data represent the mean ± SEM of three separate experiments. Unpaired Student’s t test was employed. **p*<0.05, ***p*<0.01, ****p*<0.001 and n.s. (no significant difference). According to TCGA database, the full name of each cancer type are listed as follows: BRCA (Breast invasive carcinoma), CHOL (Cholangiocarcinoma), GBM (Glioblastoma multiforme), LGG (Brain Lower Grade Glioma), HNSC (Head and Neck squamous cell carcinoma), KICH (Kidney Chromophobe), KIPAN Pan-kidney cohort (KICH+KIRC+KIRP), KIRP (Kidney renal papillary cell carcinoma), LIHC (Liver hepatocellular carcinoma), LUAD (Lung adenocarcinoma), LUSC (Lung squamous cell carcinoma), PRAD (Prostate adenocarcinoma), UCEC (Uterine Corpus Endometrial Carcinoma). (**C**) Western blot analysis reveals CD147 upregulation in HCC cell lines. 1×10^6^ cells of various cells were lysed in 200 µl RIPA buffer and mixed with 50 µl 5x SDS loading buffer before loading onto an SDS-PAGE independently. Mouse anti-Human CD147 (HIM6, Mouse IgG1) was used for western blot analysis. Anti-GAPDH was used as a loading control. (**D**) CD147 is a valid surface biomarker in HCC cell lines (SK-Hep1 and HepG2). SK-Hep1 and HepG2 (1×10^6^ cells) were stained with 2 μg FITC-mouse anti-human CD147 (anti-CD147) or 2 μg FITC-isotype mouse IgG1 (Isotype, Kappa). After incubation and washing, samples were analyzed by flow cytometry. Number represents mean fluorescence intensity (MFI) of each sample.

**Supplementary Figure 2**

**
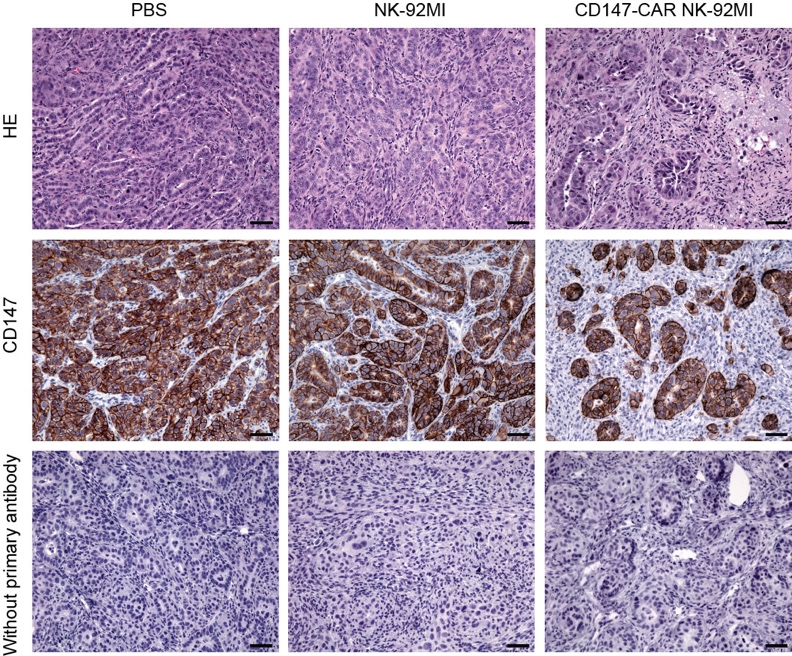
**

**Supplementary Figure 2: Histopathology analysis of CD147 antigen expression on human HCC tumor isolated from PDX mouse model.** Representative H&E and CD147 IHC staining of tumor samples from different PDX mice treated with PBS, NK-92MI, and CD147-CAR NK-92MI, respectively. Scale bars represent 50 µm. Data are representative of three independent experiments.

**Supplementary Figure 3**

**
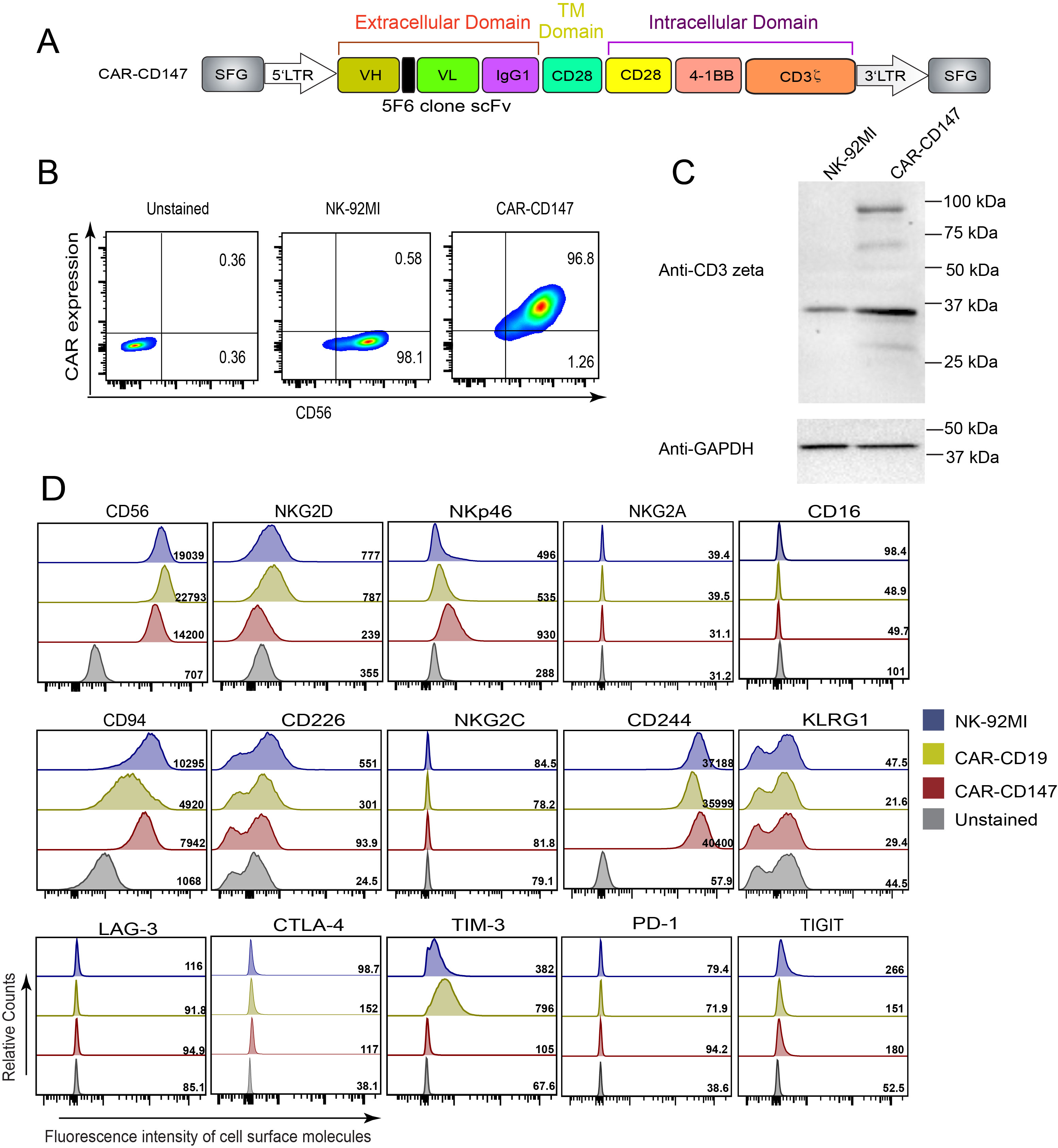
**

**Supplementary Figure 3: Schematic design of CD147-CAR and phenotyping of CAR-modified NK-92MI cells.** (**A**) Schematic design of CD147-specific CAR based on the SFG retroviral vector. The construct consisted of the CD147-specific single chain antibody fragment (scFv, clone 5F6, mIgG1), a human IgG1 CH2CH3 hinge region and CD28 transmembrane region, followed by the intracellular domains of co-stimulatory CD28, 4-1BB, and the intracellular domain of CD3ζ. (**B**) Flow cytometric analysis of CAR expression and CD56 on the surface of parental NK-92MI and CD147-CAR-NK-92MI. Data are representative of at least three experiments. (**C**) Western blot analysis of CAR expression in parental NK-92MI and CD147-CAR-NK-92MI by anti-human CD3ζ-specific antibody for detection of endogenous CD3zeta and anti-CD147-CAR fusion protein. The bands at the approximate molecular weight of around 34 to 35 kDa represent the endogenous CD3ζ protein from parental NK-92MI and CD147-CAR-NK-92MI cells. (**D**) Surface staining detecting NK activation and inhibition markers in parental NK-92MI, CAR-CD19 (4-1BB)-NK-92MI and CD147-CAR-NK-92MI. Each data represents at least three or four experiments. Number in the flow graph represents mean fluorescence intensity (MFI) of each sample.

**Supplementary Figure 4**

**
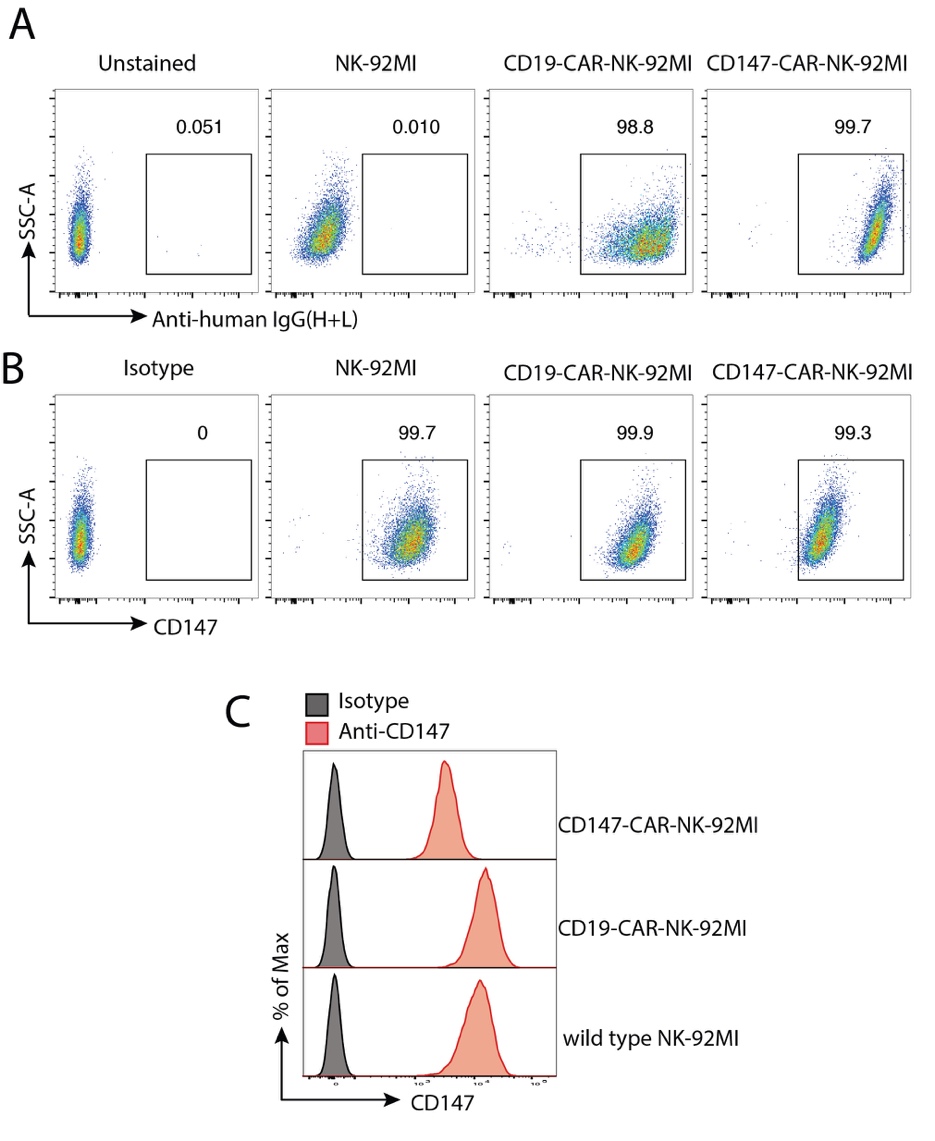
**

**Supplementary Figure 4: Flow cytometric analysis of CD147 expression on CD147-CAR-NK-92MI cells (fratricide activity analysis by flow cytometry). (A)** Flow cytometric analysis of expression of CARs on CD19-CAR-NK-92MI and CD147-CAR-NK-92MI using goat anti-human IgG (H+L). Wild type NK-92MI cells were used as control. **(B)** Flow cytometric analysis of expression of CD147 on NK-92MI, CD19-CAR-NK-92MI (control group), and CD147-CAR-NK-92MI. (**C**) Overlaid flow cytometric profile of CD147 expression levels on NK-92MI, CD19-CAR-NK-92MI, and CD147-CAR-NK-92MI. Data are representative of two independent experiments.

**Supplementary Figure 5**

**
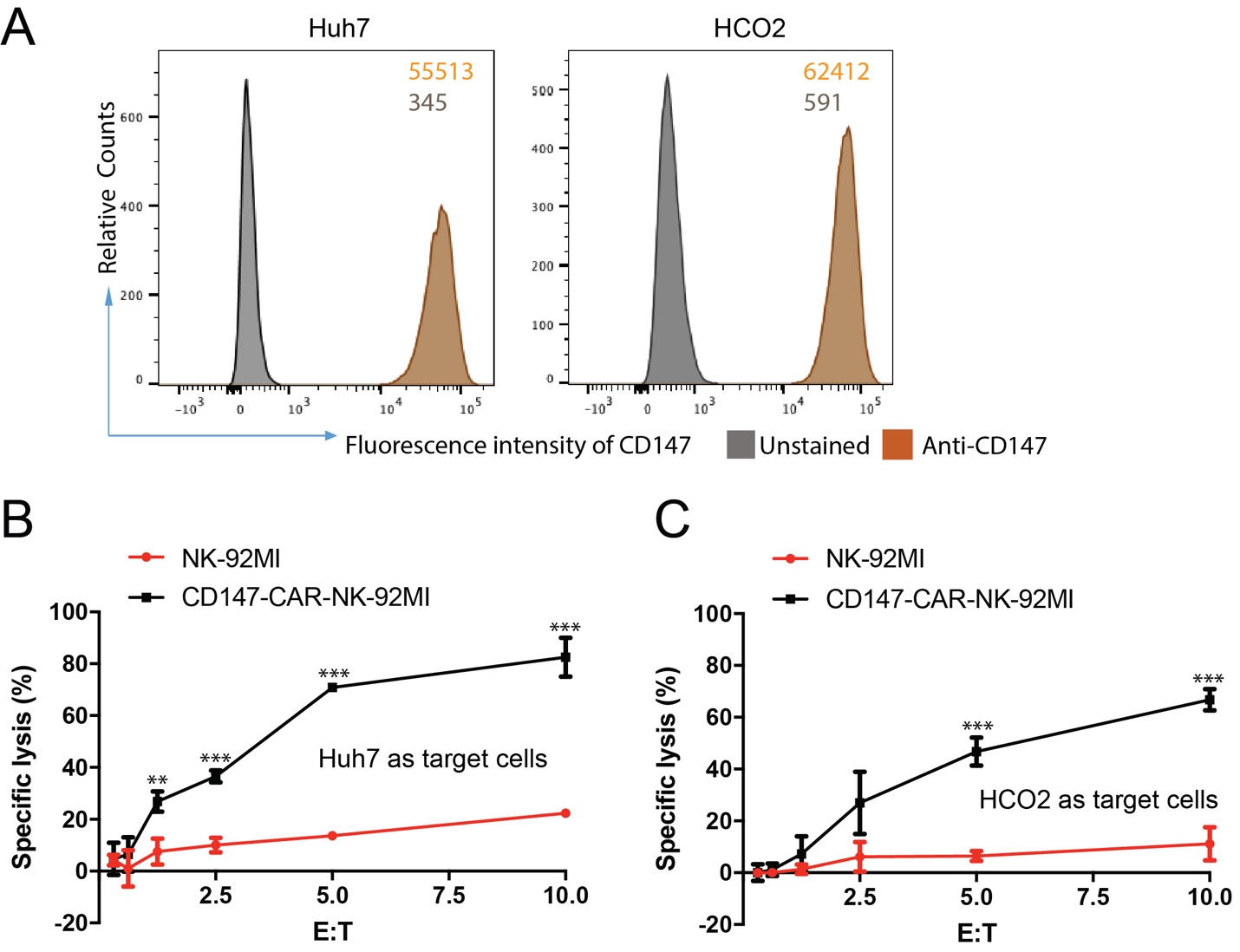
**

**Supplementary Figure 5: CD147-CAR-NK-92MI kill an additional two HCC cell lines. (A)** Representative flow cytometric staining of surface CD147 molecules on Huh7 and HCO2 cell lines. **(B)** Cytotoxicity of CD147-CAR-NK-92MI was measured by a standard 4-hr ^51^Cr release assay. CD147-positive Huh7 cells were used as the CD147-CAR-NK-92MI susceptible target cells. Wild type NK-92MI was used as control. **(C)** Cytotoxicity of CD147-CAR-NK-92MI was measured by a standard 4-hr ^51^Cr release assay. CD147-positive HCO2 cells were used as the CD147-CAR-NK-92MI susceptible target cells. Wild type NK-92MI was used as control. Data represent the mean ± SEM from three independent experiments. Unpaired Student’s t test was employed. **p*<0.05, ***p*<0.01, and ****p*<0.001.

**Supplementary Figure 6**

**
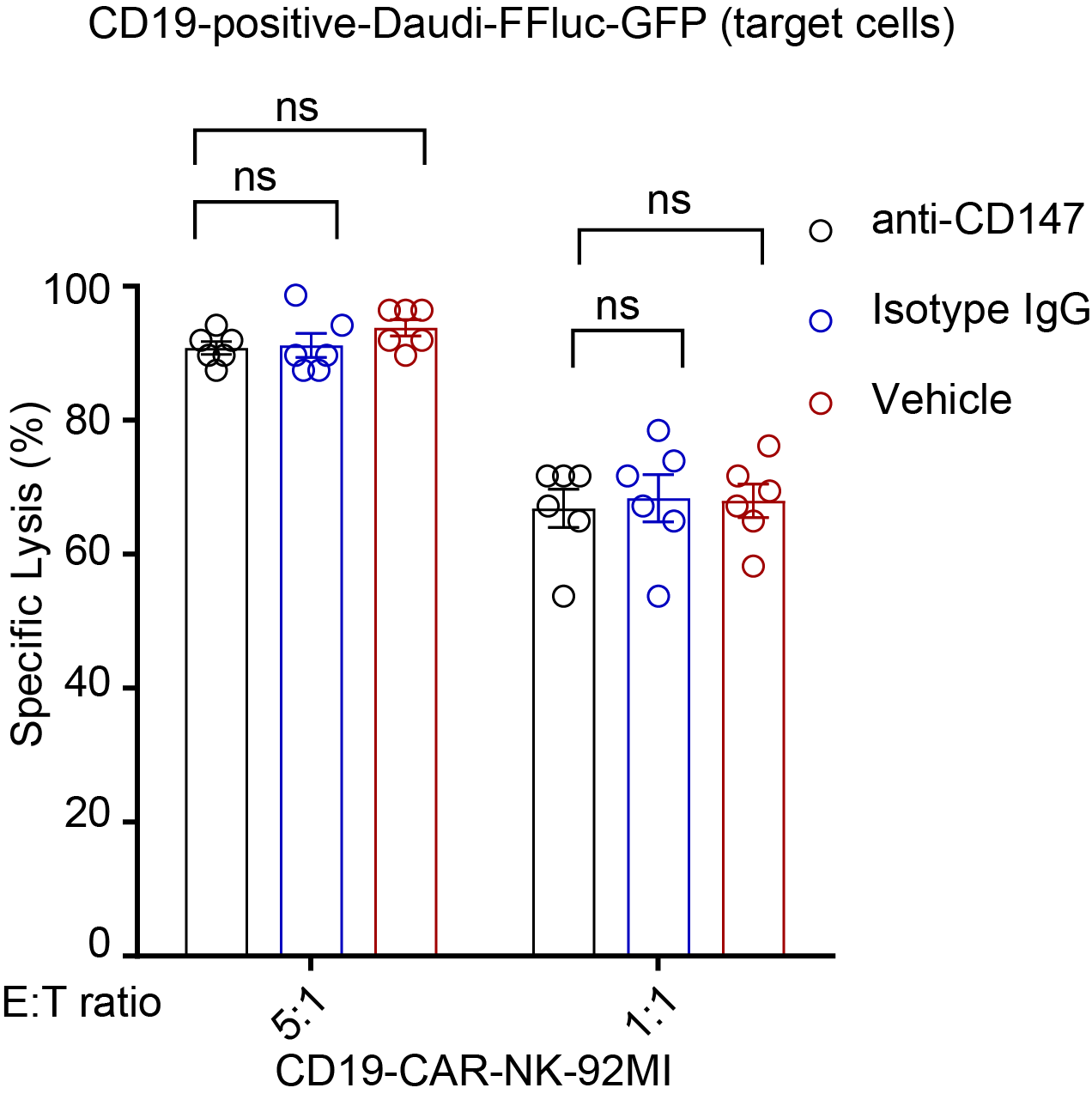
**

**Supplementary Figure 6: Mouse-anti-human CD147 (HIM6)** **cannot affect the cytotoxicity of CD19-CAR-NK cells.** Cytotoxicity of CD19-CAR-NK-92MI was measured using the FFLuc report system assay. Briefly, Daudi-FFLuc-GFP cells (1×10^4^) were pre-seeded in Matrigel (BD) treated 96-well optical-bottom microplate overnight. Effector cells (CD19-CAR-NK-92MI) at two different effector/target ratios (5:1 and 1:1, as indicated) were co-cultured for 6 hours. The luminescence signal was quantified by a microplate reader. The percentage of specific lysis was calculated. Data are pooled from three independent experiments. Error bars show ± SEM (stand error of the mean). Unpaired Student’s t test was employed. **p*<0.05, ***p*<0.01, ****p*<0.001, and n.s. (no significant difference).

**Supplementary Figure 7**

**
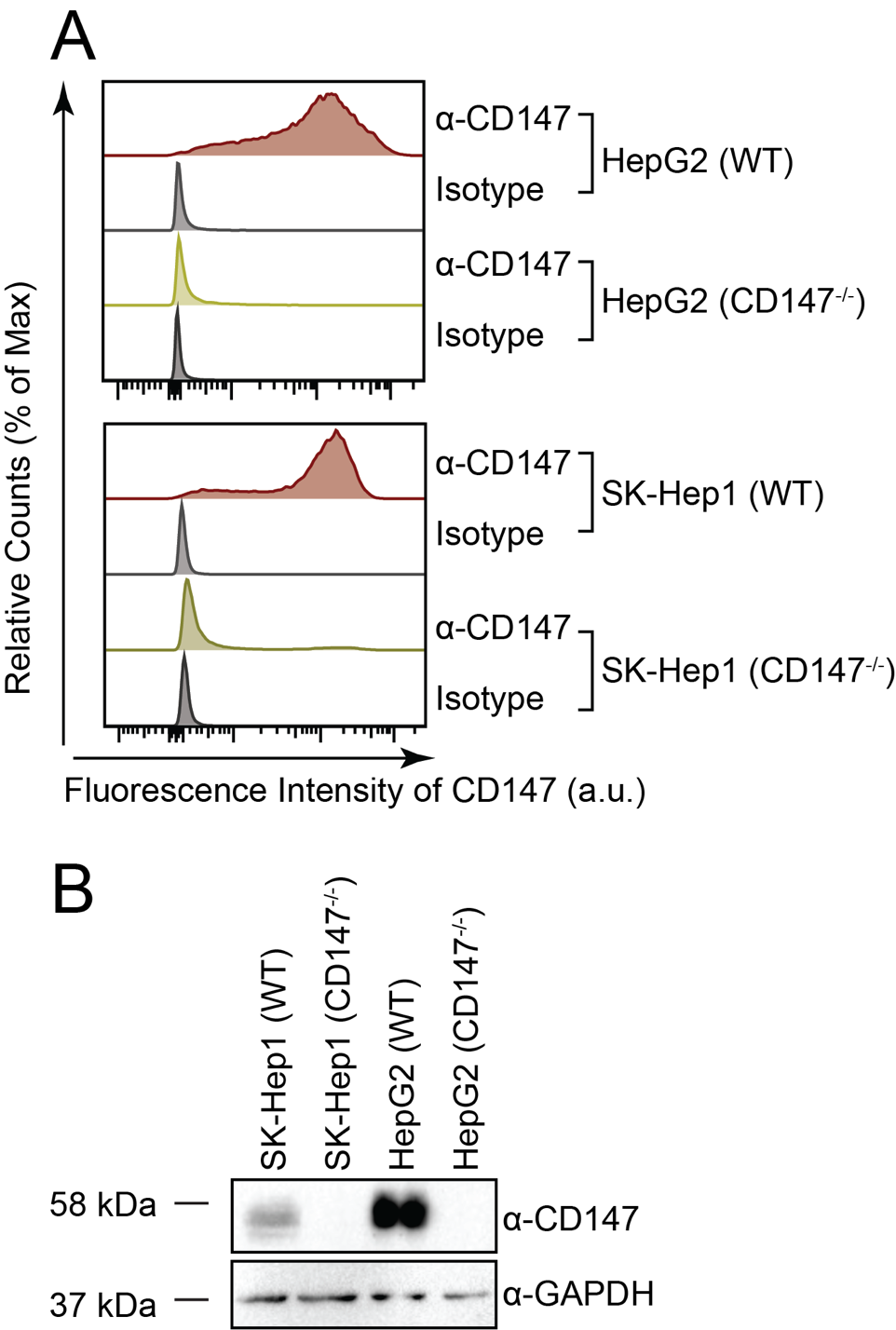
**

**Supplementary Figure 7: Verification of knockout-CD147 SK-Hep1 and HepG2 cell lines by flow cytometry and western-blot.** (**A**) Staining of surface CD147 molecules on wild-type (wt) SK-Hep1 and CD147^-/-^-SK-Hep1 cell lines (**A**, top), as well as wild-type (wt) HepG2 and CD147^-/-^-HepG2 cell lines (**A**, bottom). (**B**) Western blot analysis of CD147 molecules (~ 50 kD) on wild-type (wt) SK-Hep1 and CD147^-/-^-SK-Hep1 cell lines (**B**, left), as well as on wild-type (wt) HepG2 and CD147^-/-^-HepG2 cell lines (B, right). GAPDH was used as a loading control (**B**, bottom). Data are representative of two independent experiments.

**Supplementary Figure 8**

**
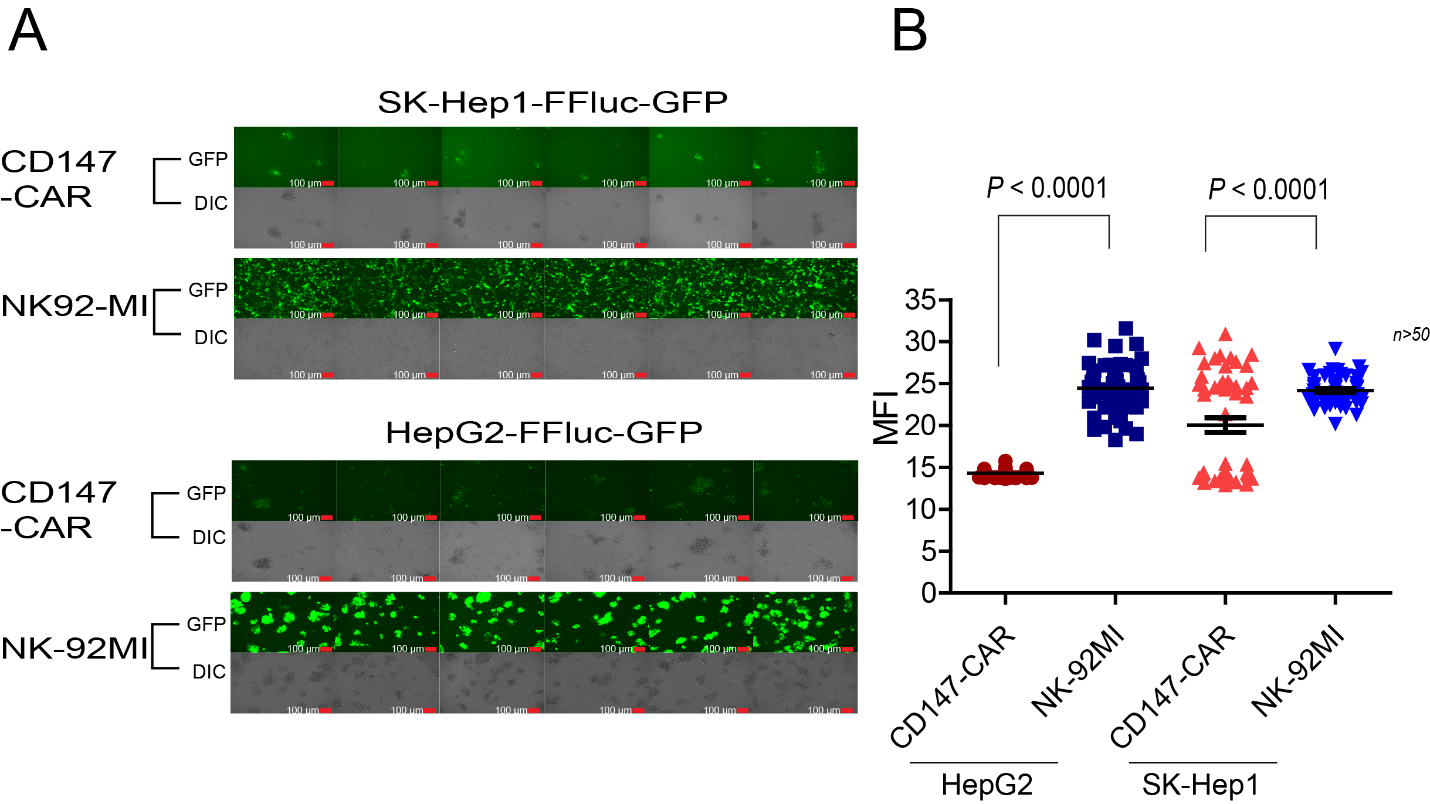
Supplementary Figure 8: Representative images of CD147-CAR-NK-92MI killing activities.** Effector cells CD147-CAR-NK-92MI or NK-92MI were co-cultured for 12 hours with target cells FFLuc-GFP-SK-Hep1 (**A**, top) and FFLuc-GFP-HepG2 (**A**, bottom) in a 96-well optical-bottom microplate. Conventional fluorescent microscopy detected GFP fluorescence (top lane) and brightfield (bottom lane) was used to visualize CD147-CAR-NK-92MI killing activities at the same setting. (**B**) The GFP fluorescence intensity was quantified using ImageJ (NIH). Data are representative of two independent experiments. All data are presented as the mean ± SEM. Unpaired Student’s t test was employed.

**Supplementary Figure 9**

**
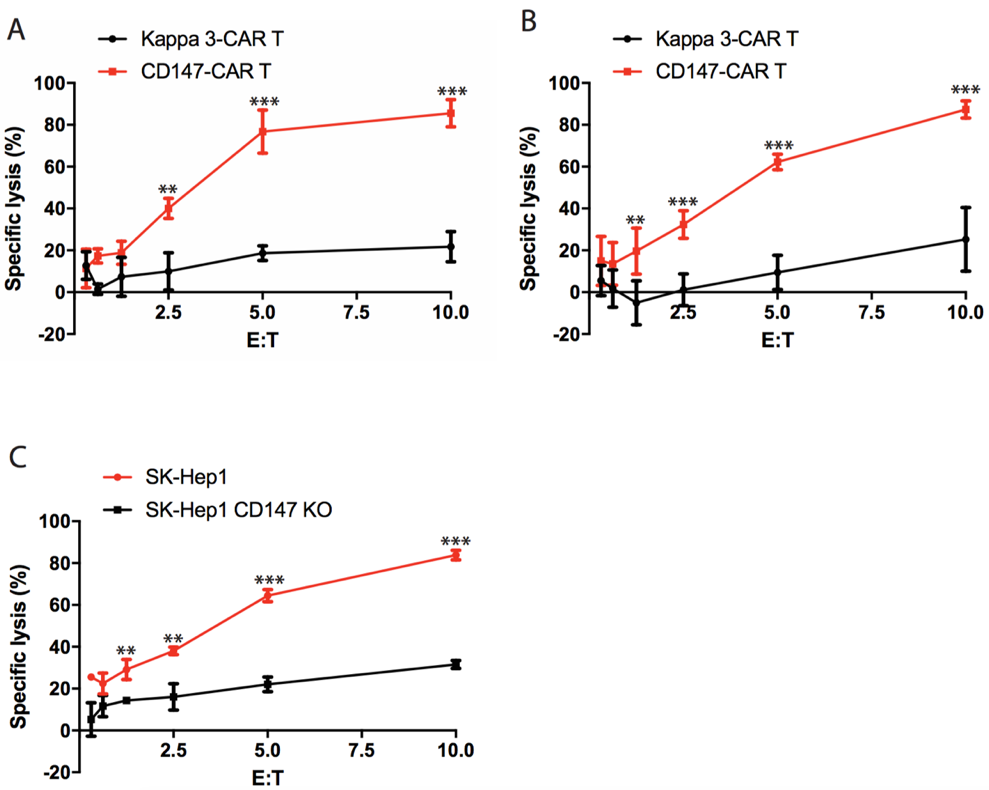
**

**Supplementary Figure 9: CD147-CAR-T cells specifically kill CD147-positive tumor cells.** (**A** and **B**) Cytotoxicity of CD147-CAR-T cells was measured by a FFLuc report system assay. CD147-positive FFLuc-GFP-HepG2 (A) and CD147-positive FFLuc-GFP-SK-Hep1 (B) were used as the CD147-CAR-T susceptible target cells. Kappa-CAR T cells were used as control groups for each experiment. (**C**) Significantly decreased cytotoxicity of CD147-CAR-T cells using CD147 knockout FFLuc-GFP-SK-Hep1 cell line by FFLuc report system assay. Briefly, effector cells (CD147-CAR-T cells) were co-cultured with target cells FFLuc-GFP-SK-Hep1 or CD147 knockout FFLuc-GFP-SK-Hep1 (1×10^4^) in a 96-well optical-bottom microplate for 6 hours. Cytotoxicity of CD147-CAR-T cells was measured by the luminescence signal read by a microplate reader. Data represent the mean ± SEM from three independent experiments. Unpaired Student’s t test was employed. **p*<0.05, ***p*<0.01, and ****p*<0.001.

**Supplementary Figure 10**

**
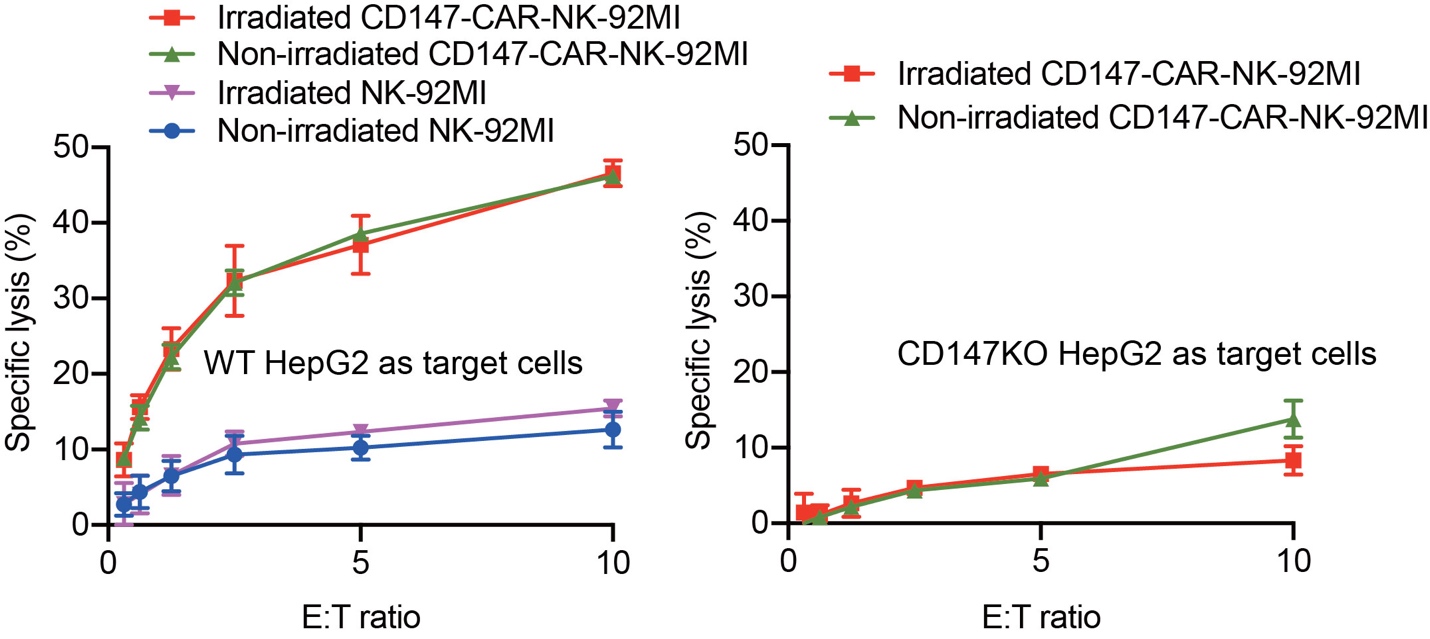
**

**Supplementary Figure 10: Comparable anti-HCC tumor activity between irradiated CD147-CAR-NK-92MI and non-irradiated CD147-CAR-NK-92MI cells in killing HCC cell lines, *in vitro*.** Cytotoxicity of irradiated (red) and non-irradiated (green) CD147-CAR-NK-92MI was measured by the standard 4-hr ^51^Cr release assay. CD147-positive wild type-HepG2 tumor cell (experimental group, left panel) or CD147-knockout (CD147KO, right panel) HepG2 tumor cell lines were used as the CD147-CAR-NK-92MI susceptible target cells. Irradiated and non-irradiated wild type NK-92MI cells were used as effector cell control groups. Data are representative of three independent experiments. All data are presented as the mean ± SEM. Unpaired Student’s t test was employed.

**Supplementary Figure 11**

**
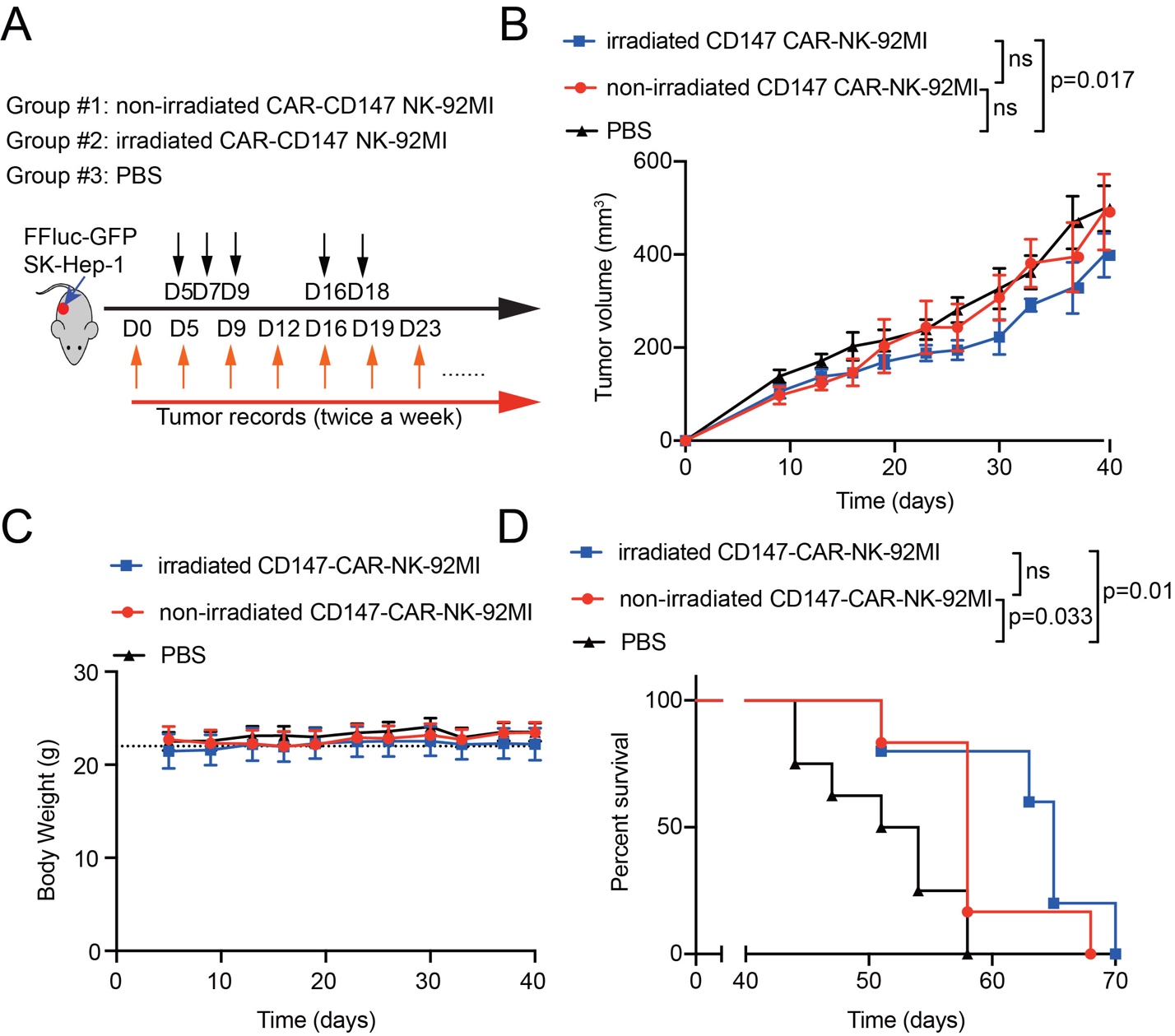
**

**Supplementary Figure 11: Comparable anti-HCC tumor activity between irradiated CD147-CAR-NK-92MI and non-irradiated CD147-CAR-NK-92MI cells in control of HCC progression in xenograft mouse model.** (**A**) Diagram of experimental design of HCC xenograft model. NSG mice were injected (s.c.) with 2 × 10^6^ SK-Hep1 cells premixed with equal volume Matrigel (Day 0). Day 0 was defined as the initial tumor cell injection time point. One day before the treatment (at day 4), tumor burden was determined (achieved nearly 50 mm^3^) and mice randomly grouped. At day 5 (D5) mice were injected (i.v.) with one dose of 1×10^7^ effector non-irradiated CD147-CAR-NK-92MI (Group #1) cells with 2×10^4^ IU IL-2. The control groups were injected with the same number of irradiated CD147-CAR-NK-92MI with 2×10^4^ IU IL-2 (Group #2) in PBS or vehicle control only (Group #3). At days 7, 9, 16, and 18, identical treatments in each group were administrated. (**B**) Quantification of tumor burden of SK-Hep1 xenografts treated with CD147-CAR-NK-92MI and PBS (vehicle control group), respectively. All results are mean ± SEM. The difference for each group was analyzed by two-way ANOVA analysis. (**C**) Quantitative body weight of each group was assessed at the indicated time points. (**D**) Kaplan–Meier survival curves of tumor-bearing mice after treatment with CD147-CAR-NK-92MI cells and PBS (vehicle control group). The p-value was analyzed by log-rank (Mantel-Cox) Test. Data are representative of two independent experiments. All data are presented as the mean ± SEM.

**Supplementary Figure 12**

**
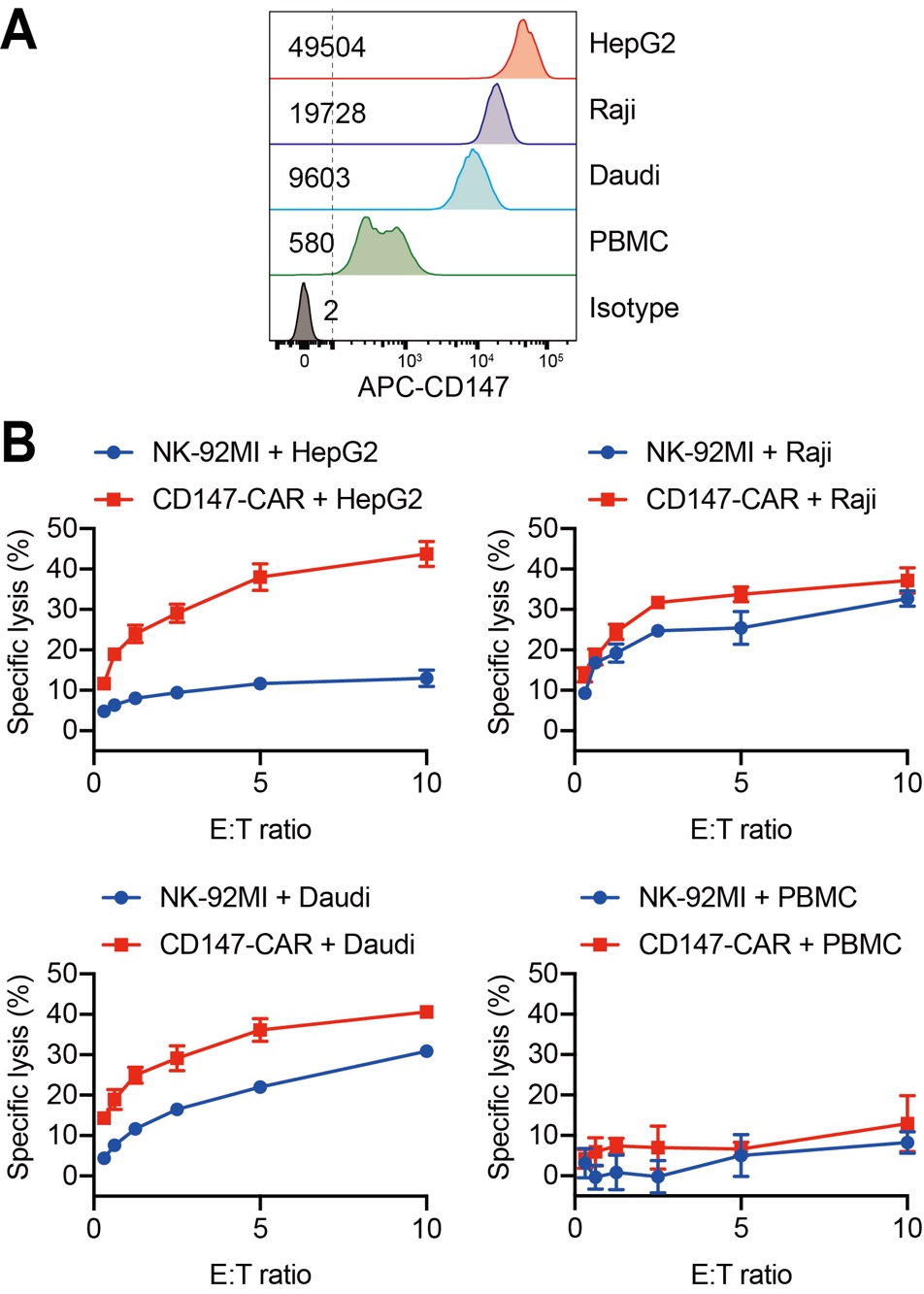
**

**Supplementary Figure 12: CD147-CAR-NK-92MI only recognize and kill high level of CD147 expression tumor cells, but no the low level of CD147 expression on hematopoietic cells. (A)** Representative flow cytometric analysis of CD147 expression on different types of cells. **(B)** Cytotoxicity of CD147-CAR-NK-92MI was measured by a standard 4-hr ^51^Cr release assay against target cells with different CD147 expression levels. Data are representative of two independent experiments. All data are presented as the mean ± SEM. Unpaired Student’s t test was employed.

**Supplementary Figure 13**


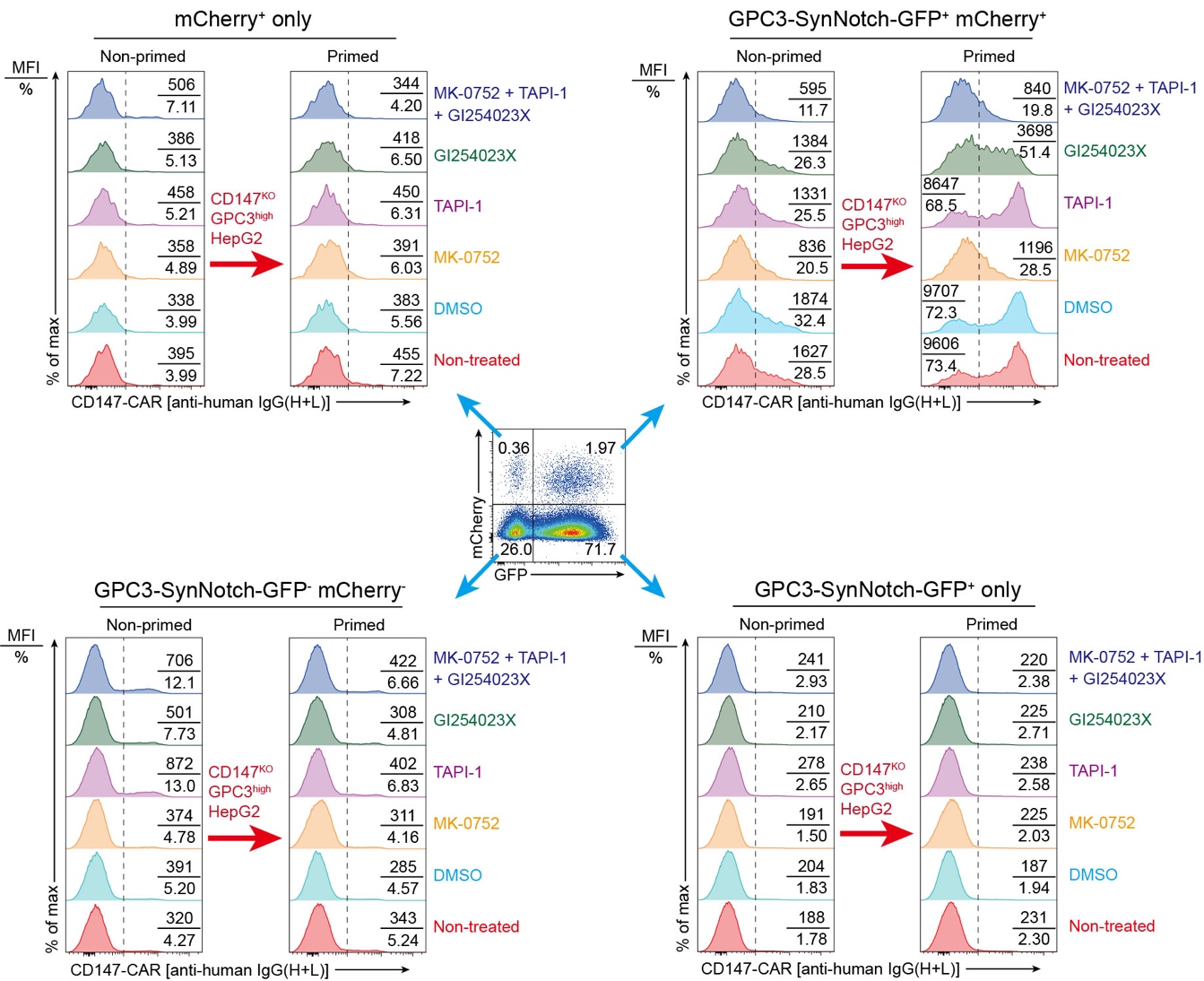


**Supplementary Figure 13: Gamma secretase inhibitor (MK-0752, a Notch signaling inhibitor) specifically blocks the GPC3-synNotch-inducible CD147-CAR expression in the GPC3-synNotch-eGFP^+^ and CD147-CAR-mCherry^+^ primary T cell subset, but not in other subsets of primary T cells.** Representative flow cytometric analysis of CD147-CAR expression among different subsets of primary T cells (middle). Transduced T cells were treated with DMSO (0.3%; control), MK-0752 (10 uM), TAPI-1 (10 uM), GI254023X (10 uM), and a combination of MK-0752 + TAPI-1 + GI254023X, respectively. Meanwhile, these cells were primed in the presence of CD147KO GPC3^high^ HepG2 cells. CD147-CAR expression on the surface of different subsets of transduced T cells was analyzed by flow cytometry. Both mean fluorescence intensity (MFI) and percentage of CD147-CAR are displayed in each representative flow cytometric chart. Data are representative of two independent experiments. All data are presented as the mean ± SEM.

**Supplementary Figure 14**


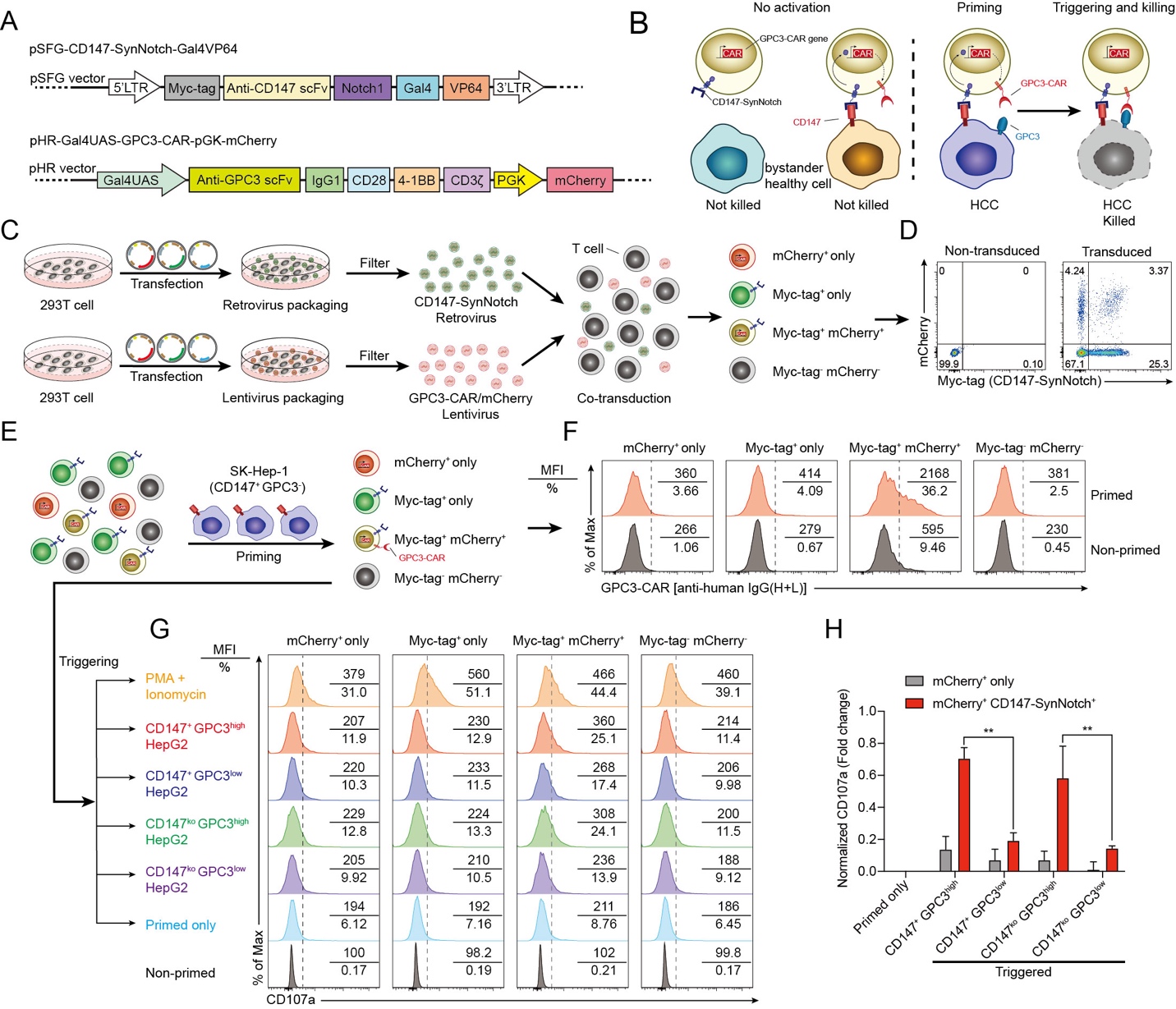


**Supplementary Figure 14: CD147-synNotch-inducible GPC3-CAR T cells selectively target GPC3^+^CD147^+^ HepG2 cells, but not GPC3^+^CD147^-^ or GPC3^-^CD147^+^ HepG2 cells. (A**) Schematic design of Myc-CD147-Gal4VP64-SynNotch receptor in the SFG retroviral vector and GPC3-CAR based on the pHR lentiviral vector. The SFG retroviral vector contains a Myc-tag, which can be used as a marker for selecting CD147-Gal4VP64-synNotch positive cells. The pHR construct consisted of the GPC3-specific single chain antibody fragment (scFv, clone 5F6, mIgG1), a human IgG1 CH2CH3 hinge region and CD28 transmembrane region, followed by the intracellular domains of co-stimulatory CD28, 4-1BB, and the intracellular domain of CD3ζ. The pHR lentiviral vector contains mCherry, which can be used as a marker for selecting cells with GPC3-CAR positive cells. (**B**) Schematic design of ‘Logic-gated’ CD147-synNotch and GPC3-CAR showing induced cytotoxicity when both antigens are co-expressed, but not activated when they are separately expressed on bystander or healthy cells. (**C** and **D**) Schematic experimental design of Myc-CD147-synNotch and GPC3-CAR-mCherry vectors co-transduced T cells (C) and Representative flow cytometric analysis of Myc-CD147-synNotch and GPC3-CAR-mCherry expression (D). (**E**) Schematic experimental design of Myc-CD147-synNotch and GPC3-CAR-mCherry vectors co-transduced in T cells, priming by GPC3^high^CD147^low^ HepG2 cell line (C), and followed by GPC3-CAR expression analysis among different subsets of transduced T cells, including mCherry positive only, GFP positive only, GFP and mCherry double positive, and GFP and mCherry double negative subsets. (**F**) Representative flow cytometric analysis of GPC3-CAR expression on the surface of different subsets of transduced T cells. Both mean fluorescence intensity (MFI) and percentage of GPC3-CAR are displayed in each representative flow cytometric chart. (**G**) Representative flow cytometric analysis of surface CD107a expression on different subsets of transduced T cells after ‘primed and triggered’ protocol by different HepG2 tumor cell lines. (**H**) Quantitative analysis of surface CD107a expression on different subsets of transduced T cells after ‘primed and triggered’ protocol by different HepG2 tumor cell lines. Fold change of CD107a MFI was calculated as follows: [(MFI_sample_ – MFI_primed only_) / MFI_primed only_]. Data are representative of two independent experiments. Unpaired Student’s t test was employed. All data are presented as the mean ± SEM. **p*<0.05, ***p*<0.01, and ****p*<0.001.

**Supplementary Figure 15**

**
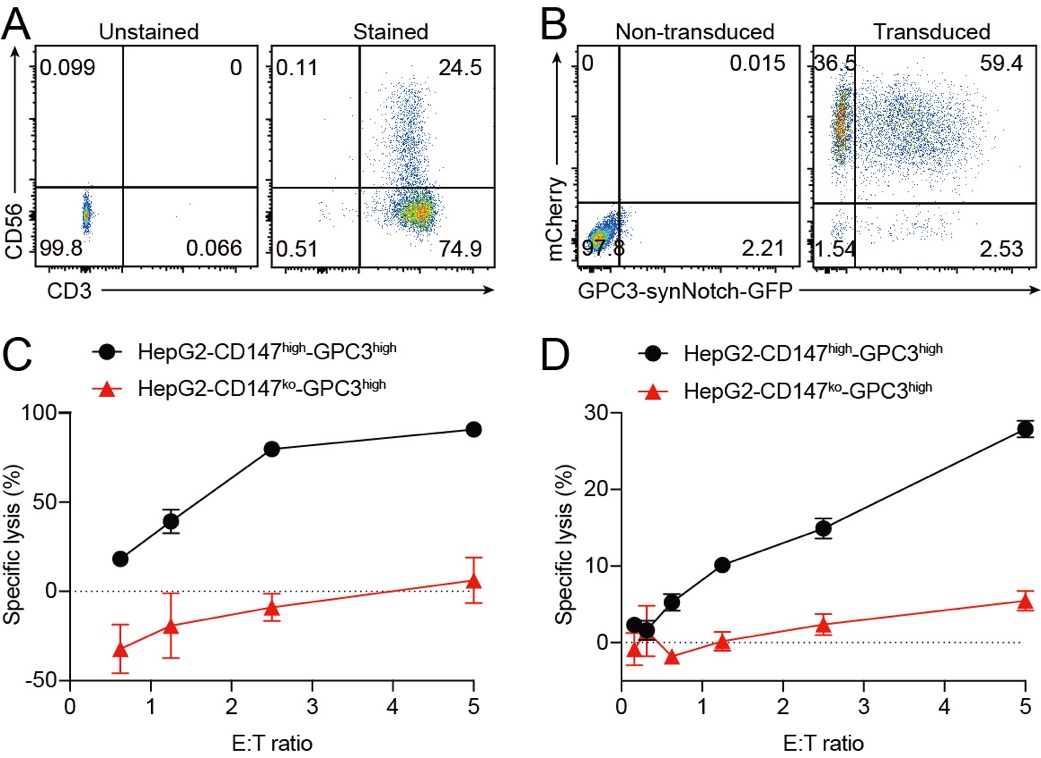
**

**Supplementary Figure 15: GPC3-synNotch-inducible CD147-CAR T cells selectively kill GPC3^high^CD147^high^ HepG2 cells but not CD147^knockout^GPC3^high^ HepG2 cells.** (**A** and **B)** Representative flow cytometric analysis of CD3, CD56, GPC3-synNotch-GFP, and CD147-CAR-mCherry expression. Primary PBMCs were transduced with CD147-CAR-mCherry lentivirus. These mCherry positive T cells were sorted using flow cytometry, followed by a secondary transduction with GPC3-synNotch-GFP retrovirus. Representative flow cytometric analysis of CD3 and CD56 (A), and GPC3-synNotch-GFP and CD147-CAR-mCherry expression (B) are displayed, respectively. (**C**) Cytotoxicity of primary GPC3-synNotch-GFP-CD147-CAR-mCherry T cells against HepG2-CD147^high^-GPC3^high^ and HepG2-CD147^ko^-GPC3^high^ was measured by 7-hour FFluc reporter assays. (**D**) Cytotoxicity of primary GPC3-synNotch-GFP-CD147-CAR-mCherry T cells against HepG2-CD147^high^-GPC3^high^ and HepG2-CD147 ^ko^-GPC3^high^ was measured by 7-hour Cr-51 release assays. Data are representative of two independent experiments. Unpaired Student’s t test was employed.

**Supplementary Figure 16**

**
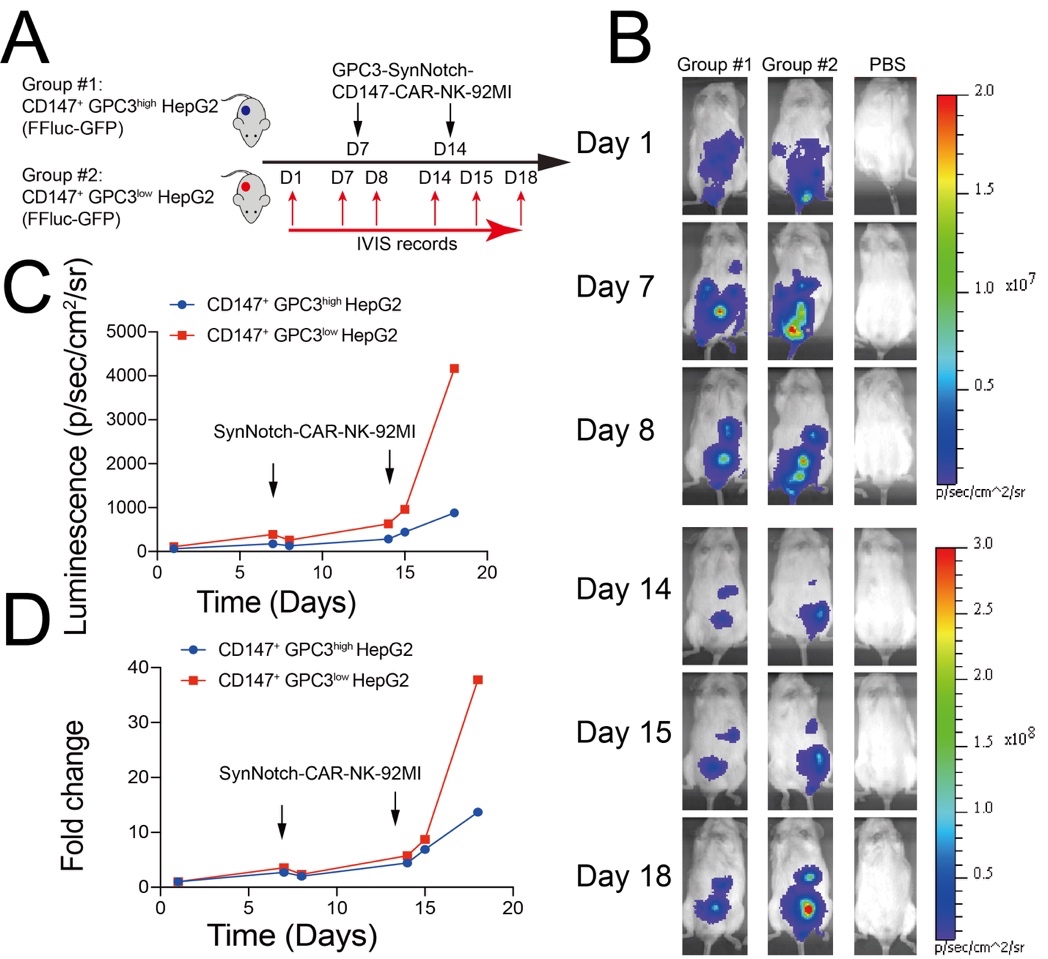
**

**Supplementary Figure 16: GPC3-synNotch- inducible CD147-CAR-NK-92MI cells control progression of CD147^positive^GPC3^high^ HepG2 xenograft NSG mouse model.** (**A**) Diagram of experimental design of HCC xenograft model. Briefly, NSG mice were intraperitoneally (i.p.) injected with 2×10^6^ CD147^+^ GPC3^high^ FFluc-GFP^+^ HepG2 cells (Group #1, n = 1) and CD147^+^ GPC3^low^ FFluc-GFP^+^ HepG2 cells (Group #2, n = 1) in 200 μL of PBS, respectively. The control groups were injected with vehicle (PBS) only. Tumor burden was monitored using IVIS at indicated days. Day 0 was defined as the initial tumor cell injection time point. At day 7 (D7) and day 14 (D14), mice were i.p. injected with 1.3 × 10^6^ irradiated GPC3-synNotch-inducible CD147-CAR-NK-92MI cells with 2×10^4^ IU IL-2. **(B)** Representative images of tumor burden at indicated days. The range of fluorescence intensity is from 2 × 10^5^ to 2 × 10^7^ units of photons/sec/cm^2^/sr for day 1, day 7, and day 8, and from 3 × 10^6^ to 3 × 10^8^ units of photons/sec/cm^2^/sr for day 14, day 15, and day 18. **(C)** Quantitative data of tumor burden at indicated time points. Mice were imaged at the indicated days to evaluate tumor burden expressed as quantified, which represent tumor growth. **(D)** Fold change of tumor burden at indicated time points normalized to the intensity measured at day 1.

**Supplementary Figure 17**


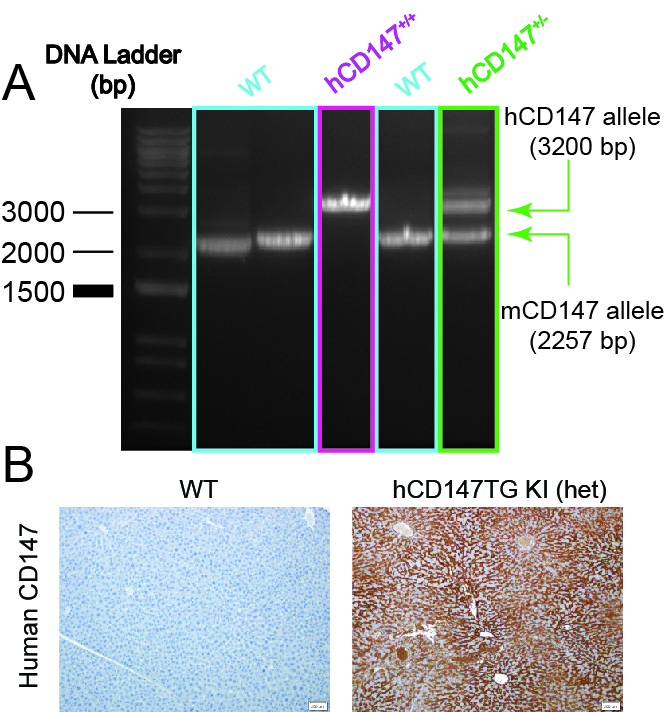


**Supplementary Figure 17:** **Genotype and IHC stain of hCD147TG mice**. (**A**) Standard PCR products imaged on a gel documentation system. (**B**) Representative IHC stain (anti-hCD147) of liver tissue from wild type (WT) and heterozygous hCD147TG mice. Scale bars represent 200 µm.

**Supplementary Figure 18**


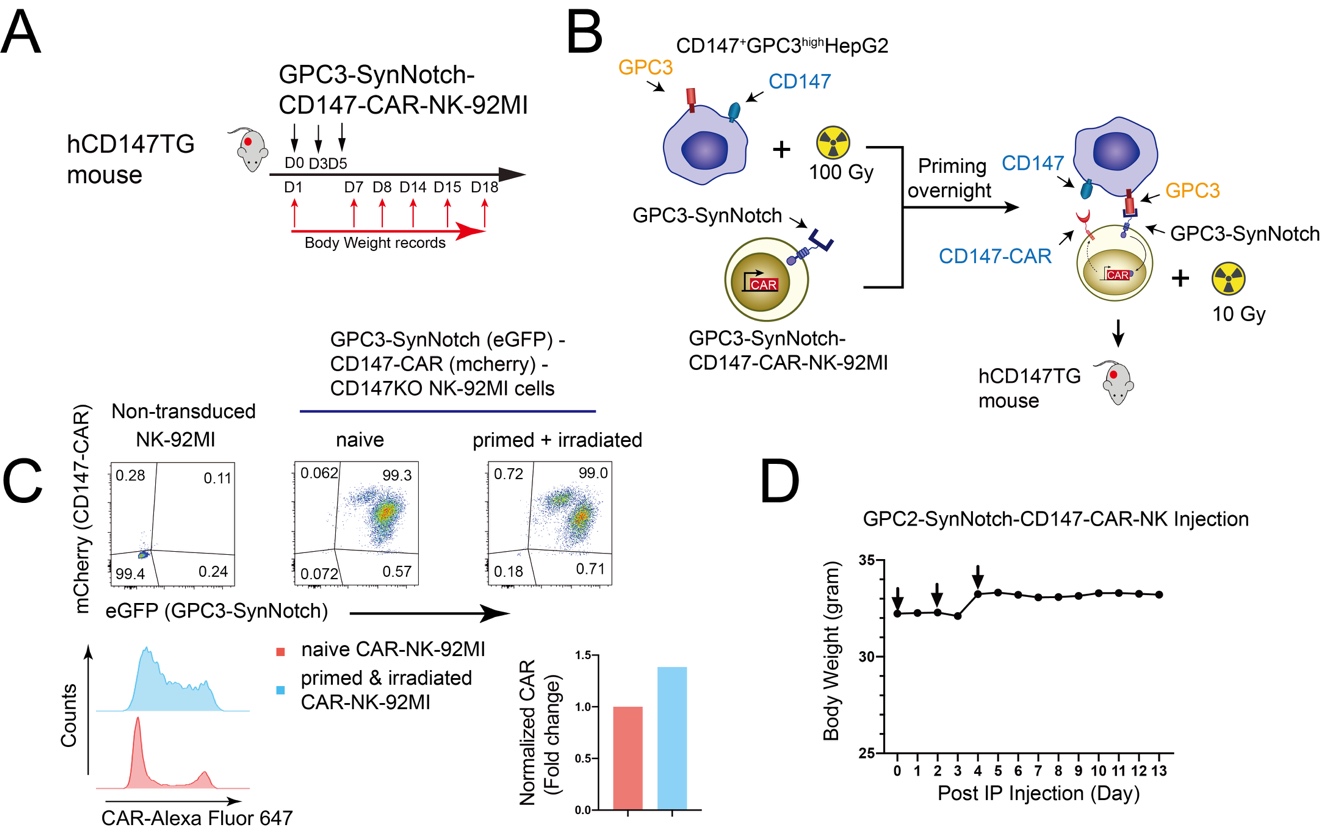


**Supplementary Figure 18:** **Activated GPC3-synNotch-inducible CD147-CAR-NK-92MI cells do not cause severe on-target/off-tumor toxicity *in vivo*.** (**A**) Diagram of experimental design of HCC in a hCD147TG transgenic mouse model. Briefly, a non-tumor-bearing hCD147TG NSG mouse was intraperitoneally (i.p.) injected with 2×10^6^ activated GPC3-synNotch-inducible CD147-CAR-NK-92MI (primed and irradiated) cells in 200 μL of PBS. Day 0 was defined as the activated GPC3-synNotch-inducible CD147-CAR-NK-92MI cell initial injection time point. **(B)** Schematic experimental design of GPC3-synNotch-inducible CD147-CAR-NK-92MI cell priming and activation by irradiated CD147^+^ GPC3^high^ FFluc-GFP^+^ HepG2 cells *in vitro*. After activation, these CD147-CAR-NK-92MI cells were irradiated before injecting into a hCD147TG mouse. **(C)** Comparison of CD147-CAR and GPC3-synNotch cell surface expressions on GPC3-synNotch-inducible CD147-CAR-NK-92MI cells after ‘primed and irradiated’ processes. (Top Row) Representative flow cytometric analysis of GPC3-synNotch-eGFP, and CD147-CAR-mCherry expression among non-transduced NK-92MI cells, naïve GPC3-synNotch-inducible CD147-CAR-NK-92MI cells, and activated GPC3-synNotch-inducible CD147-CAR-NK-92MI (primed and irradiated) cells. Bottom left panel shows increased expression level of surface CD147-CAR on activated GPC3-synNotch-inducible CD147-CAR-NK-92MI cells. Bottom right panel displays quantitative analysis of surface CD147-CAR expression on GPC3-synNotch-inducible CD147-CAR-NK-92MI cells. Fold change of CD147-CAR MFI was calculated as follows: [(MFI_primed & irradiated_ – MFI_naive_) / MFI_naive_]. (**D**) Quantitative body weight of a hCD147TG mouse was assessed at the indicated time points. Of note, the body weight slightly increased after activated GPC3-synNotch-inducible CD147-CAR-NK-92MI cell injection.

**Supplementary Table 1**: **Plasmid construction and retrovirus production**

| Construct name | Primer name | Primer sequence |
| --- | --- | --- |
| pSFG-Myc-⍺CD147-synNotch-Gal4VP64 | SFG-Myc-CD147.FOR | 5’-TGCGT CGACG AGCAG AAACT CATCT CTGAA GAGGA TCTGG AGATG AAGCT GGAAG AGAGC GGCGG-3’ |
|  | Fusion-Notch.FOR | 5’-GGCAC CAAGC TGGAG ATCAA GATCC TGGAC TACAG CTTCA CAGG-3’ |
|  | Fusion-CD147.REV | 5’-CCTGT GAAGC TGTAG TCCAG GATCT TGATC TCCAG CTTGG TGCC-3’ |
|  | SFG-Myc-CD147.REV | 5’-CTAAC GCGTT CATGA TCCGA GCATG TCCAG GTCAA AG-3’ |
| pHR-Gal4UAS-GPC3-CAR-PGK-mCherry | FP-FOR-GPC3 | 5’- TCGACATTCGTTGGATCCGCCAGCATGGAGTTTGGTTTAAGC -3’ |
|  | FP-GPC-bbz-overlap | 5’-CGGCT CCGGA ACCAA GCTGG AGATT AAGGA GCCCA AATCT CCTGA CAAAA CTCAC-3’ |
|  | RP-GPC-bbz-overlap | 5’-GTGAG TTTTG TCAGG AGATT TGGGC TCCTT AATCT CCAGC TTGGT TCCGG AGCCG-3’ |
|  | RP-REV-bbz | 5’-TAGAA TTCGT TAACC TCGAG TTAGC GAGGG GGCAG GGCCT GC-3’ |
| pSFG-Myc-⍺GPC3-synNotch-Gal4VP64-IRES-GFP | FP -NcoI | 5’-TGCCA CCATG GCAAT GGAGT TTGGT TTAAG CTGGC TGTTT TTAGT GGCCA TTTTA AAGGG CGTG-3’ |
|  | RP-MluI | 5’-CAGGA TACGC GTCTT AATCT CCAGC TTGGT TCCGG-3’ |
|  | FP-Notch-MluI | 5’-TTAAG ACGCG TATCC TGGAC TACAG CTTCA CAGGT G-3’ |
|  | RP-Notch-XhoI | 5’-TCCCG CTCGA GTCAT GATCC GAGCA TGTCC AGG-3’ |
| pHR-Gal4UAS-CD147-CAR-PGK-mCherry | FP-BamHI | 5’-TCGTT GGATC CACGC GTCGT ACGTT AATTA ACCCG GGCAT ATGTT GACTT GCGGC CGCAA C-3’ |
|  | RP-BIpI | 5’-CCATT GCTCA GCGGT GCTG-3’ |
|  | FP-MluI-147 insert | 5’-GATCC ACGCG TATGG AGTTT GGGCT GAGCT GGC-3’ |
|  | RP-NdeI-147 insert | 5’-GTCAA CATAT GTTAG CGAGG GGGCA GGGCC TGCAT G-3’ |
| pSFG-CD147-CAR-28bbz | FP-CD147  Insert | 5’-CTAGA CTGCC ATGGA GTTTG GGCTG AGCTG-3’ |
|  | RP-CD147  Insert | 5’-GACGG TGACG TACGT CTTGA TCTCC AGCTT GGTG-3’ |
