# Supplementary material for: Efficacy of anti-CD147 chimeric antigen receptors targeting hepatocellular carcinoma
Source: Nat Commun. 2020 Sep 23;11:4810. doi: 10.1038/s41467-020-18444-2 (PMC7511348; doi:10.1038/s41467-020-18444-2)
Supplement: Supplementary file 3 — Descriptions of Additional Supplementary Files [file 41467_2020_18444_MOESM3_ESM.pdf]

Descriptions of Additional Supplementary Files

Supplementary Movie 1

Description: Live imaging of CD147-CAR-NK-92MI cytotoxicity. Cytotoxicity of CD147-CAR-NK-92MI was monitored using time-lapse live fluorescence microscopy. Briefly, 1×105 GFP-SK-Hep1 cells were seeded in a 4-well chamber overnight. The next day, 2×105 CD147-CAR-NK92MI cells were added. The live imaging of CD147-CAR-NK-92MI cells was captured by a total internal reflection fluorescence microscope (TIRFM) system (Olympus) at 37°C and 5% CO_2. Images were acquired every 30 seconds for 6 hours.
